# Mapping Genetics and Epigenetics to Explore the Pathways beyond the Correlated Ageing Phenotype

**DOI:** 10.3390/genes13112169

**Published:** 2022-11-20

**Authors:** Abdelaziz Ghanemi, Mayumi Yoshioka, Jonny St-Amand

**Affiliations:** 1 Department of Molecular Medicine, Faculty of Medicine, Laval University, Québec, QC G1V 0A6, Canada; 2 Functional Genomics Laboratory, Endocrinology and Nephrology Axis, CHU de Québec-Université Laval Research Center, Québec, QC G1V 4G2, Canada

**Keywords:** ageing, genetics, epigenetics, phenotype, pathways

## Abstract

Ageing is defined by the decline in the biological and physiological functions over time, which leads to health problems and increases risks of diseases. The modern societies are characterised by an ageing population, which represents challenges for the healthcare system. Within this context, there is a need to better understand the biological mechanisms beyond ageing in order to optimise geriatric therapies and medical approaches. Herein, we suggest exploring the genetic and epigenetic patterns related to ageing and correlate them with the ageing-related phenotype of the biological entities in order to establish mechanistic links and map the molecular pathways. Such links would have diverse implications in basic research, in clinics, as well as for therapeutic studies.

In the twentieth century, human life expectancy has significantly increased [1] mainly due to the medical advances and improved healthcare human civilisation has developed [2,3]. For instance, life expectancy in the developed countries increased by around 30 years for both white males and females between 1900/1902 and 2011 [1]. It is worth noting that in the USA, for instance, there are differences in the life expectancy among racial and ethnic groups [4]. In addition, life expectancy is projected to continue to increase especially in industrialised countries [5]. Such development led to the increase in the elderly population and the healthcare needs that accompanies such change. Therefore, geriatrics—a branch of medicine focusing on elderly related pathologies and care [6,7]—acquired more importance within modern health sciences. Ageing can be defined by the effect of time on biological entities under the influence of exogenous factors, as well as the results of the endogenous reactions and processes inside the biological entity that can be a cell, a tissue, an organ, or an organism. It leads to a decline in the “biological efficacy” to perform the various biological and physiological functions required to maintain homeostasis at various levels, such as metabolism, immunity, cognition, and biomechanics. The first related fact is that ageing represents a risk factor for various diseases and health conditions, including during the ongoing COVID-19 crisis [8]. In addition, other health conditions, such as obesity, also accelerate ageing. Furthermore, the unhealthy lifestyle that characterises our era also contributes towards an unhealthy course of ageing [9,10]. Such unhealthy lifestyle habits include sedentarism and physical inactivity [11], poor diet [12], and tobacco use [13], and these increase the risk of various health problems, such as metabolic syndrome, hypertension, insulin resistance [14], cerebrovascular diseases [15], and type 2 diabetes [16,17]. Regarding the pathophysiology of ageing, this involves various processes at the subcellular level, such as oxidative stress [18,19], metabolic decline/dysfunction [20,21,22], and extracellular-matrix degradation [23]. At the functional level, ageing is associated with vascular and haemodynamic changes [15] (e.g., arterial stiffness [24], ventricular dysfunction [25] and heart failure [26]), dementia [27,28], muscle-function decline [29], respiratory-function decrease [30], skin damage [31], and bone loss [32], among other physiological changes [33,34,35,36,37].

With ageing societies, there is an urgent need to tackle age-related disorders. From one perspective, we need preventative approaches to improve healthy ageing; this requires education, sensibilisation, and probably government measures in order to encourage the general population to adapt to a healthy lifestyle in terms of physical activity, diet balance, sleeping habits, etc. The second level of action would be treatments, including therapies that target the consequences of ageing or geriatric diseases. Within this context, therapeutic targeting at the molecular and cellular levels is based on sufficient knowledge of the underlying pathways beyond the ageing phenotype. There is a need for exploration of the diverse biochemical and molecular changes seen in or resulting from ageing. Following this line of thought and considering that genomic instability is among the hallmarks of ageing [38], literature has described the ageing-related molecular features surrounding genes. Therefore, highlighting the genetic and epigenetic features characterising ageing and the correlation they have with both the ageing phenotype and geriatric symptoms would represent a strong approach to understand the mechanisms underlying ageing.

Genes’ expression patterns during ageing have been explored by genomics via various methods [39] that identified genes that are either overexpressed or downregulated with ageing. For instance, genes such as apolipoprotein D [40] and inflammation genes [40] are overexpressed with ageing, whereas other genes, including protein synthesis machinery [41] and collagen genes [40], are downregulated. Such expression patterns that change depending on ageing suggest a role of those genes either in the ageing process or, possibly, as a response (feedback) to ageing-related changes, aiming to both conserve homeostasis and prevent the biological decline.

Furthermore, while age-related genes’ expression can change depending on tissue-cell types [42], ageing-related DNA damage also has a central role in ageing [43]. Additionally, mitochondrial DNA expression also changes as genes’ encoding mitochondrial proteins are downregulated [41].

Epigenetic patterns of ageing have also been associated with ageing; these include DNA methylation [44], histone modification, chromatin remodeling, and non-coding RNAs [45]. In addition, RNA remains worth exploring considering that the dysregulation of mRNA processing is also among the hallmarks of cellular ageing [41].

Interestingly, exploring the impacts of ageing therapies (such as exercise) on the above factors in terms of expression changes is also of a particular importance and would facilitate the mapping of related therapeutic mechanisms. For instance, exploring both exercise-induced genes and genes for which the expression decreases with ageing allowed us to characterise secreted protein, acidic, and rich in cysteine (*SPARC*) as an exercise-induced gene that would counteract ageing [46,47,48]. Thus, we hypothesised that SPARC could be the molecular link via which exercise has anti-ageing effects [46]. We also suggested that, considering SPARC mediates exercise-induced benefits [49], SPARC administration could represent a substitute to exercise [50]. This is of a specific importance especially for elderly patients who are not able to perform the required physical activity due to diseases, hospitalisation, or physical disabilities [51]. This would allow them to possibly receive some of the exercise benefits although they are not able to perform exercises. Furthermore, the hypothesis pointing to SPARC as a major mediator of exercise-induced benefits is supported by the properties that have been associated with SPARC, including regeneration [52,53], anti-inflammation [54], anticancer [55], and metabolic changes [56,57], which are effects that can be obtained with exercise as well [58,59,60,61,62,63].

As an approach to plan future works to investigate ageing, the idea of this piece of writing is to explore the genetic and epigenetic changes in biological models of ageing and patients, and to compare those changes to the ageing-related phenotypes (i.e., symptoms, biological markers, etc.) seen in those same biological models of ageing and patients. This will contribute to elucidating the pathways’ underlying ageing mechanisms, which would have a variety of applications (Figure 1) in basic research, therapeutics, clinical studies, prevention, and geriatric studies. The phenotype constitutes a variety of biological patterns related to ageing or for which ageing represents a risk factor and includes muscle loss [64], atherosclerosis [65], respiratory-muscle senescence [66], the decline in learning ability [67] and the loss of proteostasis [43]. Identifying such phenotypes and their equivalent in ageing-related genomics will also contribute to a better mapping of the underlying pathways. With the recent advances, it is worth mentioning that the links between microbiota and ageing [68,69,70], as an area, are the biology of geriatrics and the question of cause-and-effect relationships has been explored [71].

Another perspective, following the same logic, is to induce such genetic and epigenetic changes in cell cultures or in animal models and observe the resulting age-related phenotypes in terms of symptoms and pathological features. For instance, free radicals are a good example for the induction of DNA damage [72] and the oxidatively induced DNA damage can be measured by mass spectrometry [73].

These illustrative examples represent the selected biological changes seen in ageing that, when combined together and/or with other markers, could also lead to an optimised biological ageing measure. Herein, the Tabula Muris Consortium published in 2020, which is a single-cell transcriptomic atlas that characterises ageing tissues in the mouse [38], represents an important set of data for the study of ageing.

Deeper exploration of changes related to ageing at the molecular and cellular levels, combined with the related phenotype, will reveal the underlying pathways of those phenotypes, identify potential therapeutic targets, optimize animal and cellular models of ageing, and evaluate the treatment approaches on these models by evaluating how such treatments impact the expression of genes, RNA profile, and epigenetics that are used as markers to evaluate biological-ageing progress and severity.

## Figures and Tables

**Figure 1:**
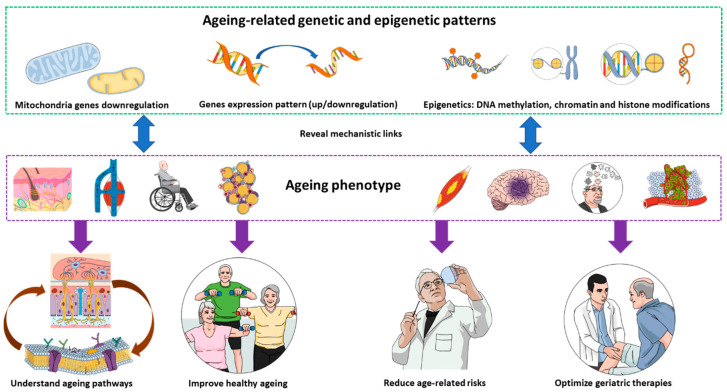
Exploring ageing-related genetic and epigenetic changes, and correlating them with ageing phenotype would have various implications in basic research, clinical studies, and geriatric healthcare.

## Data Availability

Not applicable.

## References

[1] Ulloa-Aguirre A. (2016). Preface. Rev. Investig. Clin..

[2] Aburto J.M., Villavicencio F., Basellini U., Kjærgaard S., Vaupel J.W. (2020). Dynamics of life expectancy and life span equality. Proc. Natl. Acad. Sci. USA.

[3] Vogel L. (2019). Life expectancy grows with supply of primary care doctors. CMAJ.

[4] GBD US Health Disparities Collaborators (2022). Life expectancy by county, race, and ethnicity in the USA, 2000–19: A systematic analysis of health disparities. Lancet.

[5] Kontis V., Bennett J.E., Mathers C.D., Li G., Foreman K., Ezzati M. (2017). Future life expectancy in 35 industrialised countries: Projections with a Bayesian model ensemble. Lancet.

[6] Ellis G., Sevdalis N. (2019). Understanding and improving multidisciplinary team working in geriatric medicine. Age Ageing.

[7] Voumard R., Rubli Truchard E., Benaroyo L., Borasio G.D., Büla C., Jox R.J. (2018). Geriatric palliative care: A view of its concept, challenges and strategies. BMC Geriatr..

[8] Ghanemi A., Yoshioka M., St-Amand J. (2021). Post-Coronavirus Disease-2019 (COVID-19): Toward a Severe Multi-Level Health Crisis?. Med. Sci..

[9] Södergren M. (2013). Lifestyle predictors of healthy ageing in men. Maturitas.

[10] Sowa A., Tobiasz-Adamczyk B., Topór-Mądry R., Poscia A., la Milia D.I. (2016). Predictors of healthy ageing: Public health policy targets. BMC Health Serv. Res..

[11] Arocha Rodulfo J.I. (2019). Sedentary lifestyle a disease from xxi century. Clin. Investig. Arterioscler..

[12] Petroni M.L., Brodosi L., Marchignoli F., Sasdelli A.S., Caraceni P., Marchesini G., Ravaioli F. (2021). Nutrition in Patients with Type 2 Diabetes: Present Knowledge and Remaining Challenges. Nutrients.

[13] Kushner R.F., Sorensen K.W. (2013). Lifestyle medicine: The future of chronic disease management. Curr. Opin. Endocrinol. Diabetes Obes..

[14] Saklayen M.G. (2018). The Global Epidemic of the Metabolic Syndrome. Curr. Hypertens. Rep..

[15] Beishon L., Clough R.H., Kadicheeni M., Chithiramohan T., Panerai R.B., Haunton V.J., Minhas J.S., Robinson T.G. (2021). Vascular and haemodynamic issues of brain ageing. Pflug. Arch..

[16] Shan Z., Li Y., Zong G., Guo Y., Li J., Manson J.E., Hu F.B., Willett W.C., Schernhammer E.S., Bhupathiraju S.N. (2018). Rotating night shift work and adherence to unhealthy lifestyle in predicting risk of type 2 diabetes: Results from two large US cohorts of female nurses. BMJ.

[17] Bellou V., Belbasis L., Tzoulaki I., Evangelou E. (2018). Risk factors for type 2 diabetes mellitus: An exposure-wide umbrella review of meta-analyses. PLoS ONE.

[18] Finkel T., Holbrook N.J. (2000). Oxidants, oxidative stress and the biology of ageing. Nature.

[19] Martín-Fernández B., Gredilla R. (2018). Mitochondrial oxidative stress and cardiac ageing. Clin. Investig. Arterioscler..

[20] Covarrubias A.J., Kale A., Perrone R., Lopez-Dominguez J.A., Pisco A.O., Kasler H.G., Schmidt M.S., Heckenbach I., Kwok R., Wiley C.D. (2020). Senescent cells promote tissue NAD^+^ decline during ageing via the activation of CD38^+^ macrophages. Nat. Metab..

[21] Covarrubias A.J., Perrone R., Grozio A., Verdin E. (2021). NAD^+^ metabolism and its roles in cellular processes during ageing. Nat. Rev. Mol. Cell Biol..

[22] Tarragó M.G., Chini C.C.S., Kanamori K.S., Warner G.M., Caride A., de Oliveira G.C., Rud M., Samani A., Hein K.Z., Huang R. (2018). A Potent and Specific CD38 Inhibitor Ameliorates Age-Related Metabolic Dysfunction by Reversing Tissue NAD^+^ Decline. Cell Metab..

[23] Meschiari C.A., Ero O.K., Pan H., Finkel T., Lindsey M.L. (2017). The impact of aging on cardiac extracellular matrix. Geroscience.

[24] Boutouyrie P., Chowienczyk P., Humphrey J.D., Mitchell G.F. (2021). Arterial Stiffness and Cardiovascular Risk in Hypertension. Circ. Res..

[25] de Yébenes V.G., Briones A.M., Martos-Folgado I., Mur S.M., Oller J., Bilal F., González-Amor M., Méndez-Barbero N., Silla-Castro J.C., Were F. (2020). Aging-Associated miR-217 Aggravates Atherosclerosis and Promotes Cardiovascular Dysfunction. Arterioscler. Thromb. Vasc. Biol..

[26] Li H., Hastings M.H., Rhee J., Trager L.E., Roh J.D., Rosenzweig A. (2020). Targeting Age-Related Pathways in Heart Failure. Circ. Res..

[27] Grande G., Qiu C., Fratiglioni L. (2020). Prevention of dementia in an ageing world: Evidence and biological rationale. Ageing Res. Rev..

[28] Fratiglioni L., Marseglia A., Dekhtyar S. (2020). Ageing without dementia: Can stimulating psychosocial and lifestyle experiences make a difference?. Lancet Neurol..

[29] Izquierdo M., Merchant R.A., Morley J.E., Anker S.D., Aprahamian I., Arai H., Aubertin-Leheudre M., Bernabei R., Cadore E.L., Cesari M. (2021). International Exercise Recommendations in Older Adults (ICFSR): Expert Consensus Guidelines. J. Nutr. Health Aging.

[30] Janssens J.P., Pache J.C., Nicod L.P. (1999). Physiological changes in respiratory function associated with ageing. Eur. Respir. J..

[31] Bonté F., Girard D., Archambault J.C., Desmoulière A. (2019). Skin Changes during Ageing. Subcell Biochem..

[32] Roberts S., Colombier P., Sowman A., Mennan C., Rölfing J.H., Guicheux J., Edwards J.R. (2016). Ageing in the musculoskeletal system. Acta Orthop..

[33] Salvi S.M., Akhtar S., Currie Z. (2006). Ageing changes in the eye. Postgrad. Med. J..

[34] Aguayo-Mazzucato C. (2020). Functional changes in beta cells during ageing and senescence. Diabetologia.

[35] Juan S.M.A., Adlard P.A. (2019). Ageing and Cognition. Subcell Biochem..

[36] Stern Y. (2012). Cognitive reserve in ageing and Alzheimer’s disease. Lancet Neurol..

[37] van den Beld A.W., Kaufman J.M., Zillikens M.C., Lamberts S.W.J., Egan J.M., van der Lely A.J. (2018). The physiology of endocrine systems with ageing. Lancet Diabetes Endocrinol..

[38] The Tabula Muris Consortium (2020). A single-cell transcriptomic atlas characterizes ageing tissues in the mouse. Nature.

[39] Melouane A., Ghanemi A., Aubé S., Yoshioka M., St-Amand J. (2018). Differential gene expression analysis in ageing muscle and drug discovery perspectives. Ageing Res. Rev..

[40] de Magalhães J.P., Curado J., Church G.M. (2009). Meta-analysis of age-related gene expression profiles identifies common signatures of aging. Bioinformatics.

[41] Frenk S., Houseley J. (2018). Gene expression hallmarks of cellular ageing. Biogerontology.

[42] Zhang M.J., Pisco A.O., Darmanis S., Zou J. (2021). Mouse aging cell atlas analysis reveals global and cell type-specific aging signatures. Elife.

[43] Schumacher B., Pothof J., Vijg J., Hoeijmakers J.H.J. (2021). The central role of DNA damage in the ageing process. Nature.

[44] Morgan A.E., Davies T.J., Mc Auley M.T. (2018). The role of DNA methylation in ageing and cancer. Proc. Nutr. Soc..

[45] Ghanemi A., Yoshioka M., St-Amand J. (2021). Ageing and Obesity Shared Patterns: From Molecular Pathogenesis to Epigenetics. Diseases.

[46] Ghanemi A., Melouane A., Yoshioka M., St-Amand J. (2022). Secreted Protein Acidic and Rich in Cysteine (Sparc) KO Leads to an Accelerated Ageing Phenotype Which Is Improved by Exercise Whereas SPARC Overexpression Mimics Exercise Effects in Mice. Metabolites.

[47] Ghanemi A., Yoshioka M., St-Amand J. (2021). Measuring Exercise-Induced Secreted Protein Acidic and Rich in Cysteine Expression as a Molecular Tool to Optimize Personalized Medicine. Genes.

[48] Kwon J.H., Moon K.M., Min K.W. (2020). Exercise-Induced Myokines can Explain the Importance of Physical Activity in the Elderly: An Overview. Healthcare.

[49] Ghanemi A., Melouane A., Yoshioka M., St-Amand J. (2020). Exercise Training of Secreted Protein Acidic and Rich in Cysteine (Sparc) KO Mice Suggests That Exercise-Induced Muscle Phenotype Changes Are SPARC-Dependent. Appl. Sci..

[50] Ghanemi A., Yoshioka M., St-Amand J. (2022). Genetic Expression between Ageing and Exercise: Secreted Protein Acidic and Rich in Cysteine as a Potential “Exercise Substitute” Antiageing Therapy. Genes.

[51] Ghanemi A., Yoshioka M., St-Amand J. (2022). Secreted Protein Acidic and Rich in Cysteine as an Exercise-Induced Gene: Towards Novel Molecular Therapies for Immobilization-Related Muscle Atrophy in Elderly Patients. Genes.

[52] Ghanemi A., Yoshioka M., St-Amand J. (2021). Secreted Protein Acidic and Rich in Cysteine as A Regeneration Factor: Beyond the Tissue Repair. Life.

[53] Ghanemi A., Yoshioka M., St-Amand J. (2021). Secreted Protein Acidic and Rich in Cysteine as a Molecular Physiological and Pathological Biomarker. Biomolecules.

[54] Ghanemi A., Yoshioka M., St-Amand J. (2020). Secreted protein acidic and rich in cysteine and inflammation: Another homeostatic property?. Cytokine.

[55] Ghanemi A., Yoshioka M., St-Amand J. (2020). Secreted protein acidic and rich in cysteine and cancer: A homeostatic hormone?. Cytokine.

[56] Ghanemi A., Melouane A., Yoshioka M., St-Amand J. (2019). Secreted protein acidic and rich in cysteine and bioenergetics: Extracellular matrix, adipocytes remodeling and skeletal muscle metabolism. Int. J. Biochem. Cell Biol..

[57] Ghanemi A., Yoshioka M., St-Amand J. (2020). Secreted Protein Acidic and Rich in Cysteine: Metabolic and Homeostatic Properties beyond the Extracellular Matrix Structure. Appl. Sci..

[58] Sandrow-Feinberg H.R., Houlé J.D. (2015). Exercise after spinal cord injury as an agent for neuroprotection, regeneration and rehabilitation. Brain Res..

[59] Petersen A.M., Pedersen B.K. (2005). The anti-inflammatory effect of exercise. J. Appl. Physiol..

[60] Scheffer D.D.L., Latini A. (2020). Exercise-induced immune system response: Anti-inflammatory status on peripheral and central organs. Biochim. Biophys. Acta Mol. Basis Dis..

[61] Hojman P., Gehl J., Christensen J.F., Pedersen B.K. (2018). Molecular Mechanisms Linking Exercise to Cancer Prevention and Treatment. Cell Metab..

[62] Swift D.L., McGee J.E., Earnest C.P., Carlisle E., Nygard M., Johannsen N.M. (2018). The Effects of Exercise and Physical Activity on Weight Loss and Maintenance. Prog. Cardiovasc. Dis..

[63] Martin K.S., Azzolini M., Lira Ruas J. (2020). The kynurenine connection: How exercise shifts muscle tryptophan metabolism and affects energy homeostasis, the immune system, and the brain. Am. J. Physiol. Cell Physiol..

[64] Curtis E., Litwic A., Cooper C., Dennison E. (2015). Determinants of Muscle and Bone Aging. J. Cell Physiol..

[65] Uryga A.K., Bennett M.R. (2016). Ageing induced vascular smooth muscle cell senescence in atherosclerosis. J. Physiol..

[66] Gea J., Ausín P., Martínez-Llorens J.M., Barreiro E. (2020). Respiratory muscle senescence in ageing and chronic lung diseases. Eur. Respir. Rev..

[67] Albert M.S. (1997). The ageing brain: Normal and abnormal memory. Philos. Trans. R. Soc. Lond. B Biol. Sci..

[68] Maynard C., Weinkove D. (2018). The Gut Microbiota and Ageing. Subcell Biochem..

[69] Brunt V.E., Gioscia-Ryan R.A., Richey J.J., Zigler M.C., Cuevas L.M., Gonzalez A., Vázquez-Baeza Y., Battson M.L., Smithson A.T., Gilley A.D. (2019). Suppression of the gut microbiome ameliorates age-related arterial dysfunction and oxidative stress in mice. J. Physiol..

[70] Kim S., Jazwinski S.M. (2018). The Gut Microbiota and Healthy Aging: A Mini-Review. Gerontology.

[71] Clark R.I., Walker D.W. (2018). Role of gut microbiota in aging-related health decline: Insights from invertebrate models. Cell Mol. Life Sci..

[72] Dizdaroglu M., Jaruga P. (2012). Mechanisms of free radical-induced damage to DNA. Free Radic. Res..

[73] Dizdaroglu M., Coskun E., Jaruga P. (2015). Measurement of oxidatively induced DNA damage and its repair, by mass spectrometric techniques. Free Radic. Res..

